# Diabetes Prevalence in Relation to Serum Concentrations of Polychlorinated Biphenyl (PCB) Congener Groups and Three Chlorinated Pesticides in a Native American Population

**DOI:** 10.1289/ehp.1509902

**Published:** 2016-04-01

**Authors:** Zafar Aminov, Richard Haase, Robert Rej, Maria J. Schymura, Azara Santiago-Rivera, Gayle Morse, Anthony DeCaprio, David O. Carpenter, the Akwesasne Task Force on the Environment

**Affiliations:** 1 Department of Environmental Health Sciences, School of Public Health, and; 2 Institute for Health and the Environment, University at Albany, State University of New York (SUNY), Rensselaer, New York, USA; 3 Department of Counseling Psychology, University at Albany, SUNY, Albany, New York, USA; 4 Wadsworth Center, New York State Department of Health and Department of Biomedical Sciences, School of Public Health, University at Albany, SUNY, Rensselaer, New York, USA; 5 Department of Epidemiology and Biostatistics, School of Public Health, University at Albany, SUNY, Rensselaer, New York, USA; 6 Mohawk Nation at Akwesasne, Hogansburg, New York, USA

## Abstract

**Background:**

Exposure to persistent organic pollutants (POPs) is known to increase risk of diabetes.

**Objective:**

To determine which POPs are most associated with prevalence of diabetes in 601 Akwesasne Native Americans.

**Methods:**

Multiple logistic regression analysis was used to assess associations between quartiles of concentrations of 101 polychlorinated biphenyl (PCBs) congeners, congener groups and three chlorinated pesticides [dichlorodiphenyldichloroethylene (DDE), hexachlorobenzene (HCB) and mirex] with diabetes. In Model 1, the relationship between quartiles of exposure and diabetes were adjusted only for sex, age, body mass index (BMI), and total serum lipids. Model 2 included additional adjustment for either total PCBs or total pesticides.

**Results:**

Total serum PCB and pesticide concentrations were each significantly associated with prevalence of diabetes when adjusted only for covariates (Model 1), but neither showed a significant OR for highest to lowest quartiles after additional adjustment for the other (Model 2). When applying Model 2 to PCB congener groups and individual pesticides, there were significant omnibus differences between the four quartiles (all ps < 0.042) for most groups, with the exception of penta- and hexachlorobiphenyls, DDE and mirex. However, when comparing highest to lowest quartiles only non- and mono-ortho PCBs [OR = 4.55 (95% CI: 1.48, 13.95)], tri- and tetrachloro PCBs [OR = 3.66 (95% CI: 1.37, –9.78)] and HCB [OR = 2.64 (95% CI: 1.05, 6.61)] showed significant associations with diabetes. Among the non- and mono-ortho congeners, highest to lowest quartile of dioxin TEQs was not significant [OR = 1.82 (95% CI: 0.61, 5.40)] but the OR for the non-dioxin-like congeners was [OR = 5.01 (95% CI: 1.76, 14.24)].

**Conclusion:**

The associations with diabetes after adjustment for other POPs were strongest with the more volatile, non-dioxin-like, low-chlorinated PCB congeners and HCB. Because low-chlorinated congeners are more volatile, these observations suggest that inhalation of vapor-phase PCBs is an important route of exposure.

**Citation:**

Aminov Z, Haase R, Rej R, Schymura MJ, Santiago-Rivera A, Morse G, DeCaprio A, Carpenter DO, and the Akwesasne Task Force on the Environment. 2016. Diabetes prevalence in relation to serum concentrations of polychlorinated biphenyl (PCB) congener groups and three chlorinated pesticides in a Native American population. Environ Health Perspect 124:1376–1383; http://dx.doi.org/10.1289/ehp.1509902

## Introduction

Diabetes is a very prevalent chronic disease in developed countries and is increasing in incidence around the world, with a significant burden of morbidity and mortality as well as health care costs (CDC 2014). Diabetes is an important risk factor for coronary and peripheral vascular disease, diabetic retinopathy, kidney disease, and nervous system impairment. In 2010 alone there were approximately 1.9 million new diagnoses of diabetes in Americans 20 years of age and older (CDC 2014), and data from the Framingham Heart Study indicates a doubling of incidence of type 2 diabetes in the last 30 years (Fox et al. 2006). Incidence and prevalence of diabetes vary by age, race, lifestyle, and socioeconomic factors and are higher among Native Americans than among Caucasians (Acton et al. 2002). Known risk factors for diabetes include obesity, genetic predisposition, hyperinsulinemia (a marker for insulin resistance), sedentary lifestyle (Hu et al. 2001; Kriska et al. 2003) and cigarette smoking (Rimm et al. 1995; Will et al. 2001).

Polychlorinated biphenyls (PCBs) were produced for various uses until the late 1970s when their production was banned in the United States (ATSDR 2000). Large quantities of PCBs have been released into the environment. They are persistent substances both in the environment and in living organisms, and they bioaccumulate and biomagnify in the food chain. Once in the human body they persist for long periods, accumulating in adipose tissue and in the lipid component of serum. Organochlorine pesticides such as dichlorodiphenyltrichloroethane (DDT) and its major metabolite, dichlorodiphenyldichloroethylene (DDE), and hexachlorobenzene (HCB) and mirex are also banned in most countries (Dunlap 1981; ATSDR 1995), but are persistent and present in the environment and in human serum (Carpenter 2006).

The Mohawk Nation at Akwesasne is a Native American population residing along the St. Lawrence River that separates New York State from the provinces of Ontario and Quebec. Mohawks are a traditional fish-eating community. There were three aluminum foundries just upriver from the reservation, operated by General Motors, Reynold Metals, and ALCOA (Hwang et al. 1993). PCBs (primary Aroclor 1248) were used as hydraulic fluids at all three facilities. PCBs that leaked were washed into the St. Lawrence River and its tributaries. In previous studies, PCB levels in serum and in the breast milk of Mohawk women were correlated to rates of consumption of local fish, although rates of local fish consumption have declined after issuance of advisories (Hwang et al. 1996; Fitzgerald et al. 2004).

Recent studies have reported an association between exposure to persistent organic pollutants (POPs) and diabetes (reviewed by Carpenter 2008). Using data from the National Health and Nutrition Examination Survey (NHANES), Lee et al. (2006) found a dose–response relationship between serum concentrations of six persistent POPs (PCB153, two dioxin congeners, oxychlordane, DDE, and *trans*-nonachlor) and diabetes. There has, however, been difficulty in determining whether specific POPs are responsible for the associations seen since all of the ones usually studied are lipophilic and migrate together. Thus, finding an association between levels of one lipophilic chemical and diabetes, if there is no control for concentrations of other lipophilic chemicals, does not necessarily suggest a cause and effect relationship with the first chemical but may rather reflect actions of another chemical whose concentration correlates with the first one. In an earlier publication, Aminov et al. (2013)presented a detailed analysis of the benefits and dangers of adjusting for concentrations of individual lipophilic POPs. In the present data analysis, we have applied two models to the data with increasing levels of adjustment in order to distinguish which of the PCB congener groups and which of the pesticides are most closely associated with elevations in diabetes. Because our PCB analytical laboratory monitors 101 congeners and DDE, HCB, and mirex, we have greater ability than most to determine differences in actions of the various congener groups and pesticides.

A preliminary study of associations between exposure to these chemicals and diabetes incidence has been reported in a subset of adult Mohawks (Codru et al. 2007). This study expands the analysis to 601 Mohawks > 18 years of age and reports the associations with total PCBs, PCB congener groups, total pesticides, and three individual pesticides after adjusting for demographic covariates and before and after adjustment for other POPs.

## Methods

The study population consisted of 601 Mohawk adults 18–84 years old recruited between 1995 and 2000 for whom complete data was available on serum concentration of 101 PCB congeners and 3 pesticides (DDE, HCB, and mirex), as well as age, sex, height, and weight. Mohawk staff members supported by the grant provided a listing of all households on the reserve, and a random sample of households was selected. One person per household was invited to participate provided they agreed to provide information and a blood sample. Details about the study design and selection of participants have been described in the previous publications that have dealt with subsets of the total study population (Codru et al. 2007; Goncharov et al. 2008; Santiago-Rivera et al. 2007). Human subjects approval was obtained from the institutional review boards of both the University at Albany, State University of New York (SUNY) and the New York State Department of Health.

Written informed consent was obtained from all participants. Participants were then administered a core interview that included demographic information, questions on diet, residential and occupational exposures, and education. The standardized questionnaire, administered by an in-person interview, included open-ended questions on participants’ height, weight, medical conditions, and medications.

Blood samples (5 mL for glucose and lipids and 10 mL for PCBs and pesticides) were obtained by venipuncture between 0730 and 1030 hours after fasting overnight. Serum glucose (Kunst et al. 1984), cholesterol (Allain et al. 1974), and triglyceride (Kohlmeier 1986) concentrations were measured in the New York State Department of Health Wadsworth Center on a Hitachi 911 analyzer (Roche Diagnostics, Indianapolis, IN). The facility is Clinical Laboratory Improvement Amendments approved and, at the time of the study, was a member of the Centers for Disease Control and Prevention (CDC) reference laboratory network for lipid measurements. Total serum lipids were estimated as described by Phillips et al. (1989) from the following formula (Equation 1):

Total lipids (mg/dL) = 2.27 Total cholesterol + Triglycerides + 0.623. [1]

PCB analysis was performed in the Exposure Assessment Laboratory, University at Albany, SUNY as described by DeCaprio et al. (2000). These ultratrace analytical methods utilize dual-column gas chromatography with electron-capture detection to measure 92 analytical peaks that represent 83 single PCB congeners and 18 congeners as pairs or triplets, for a total of 101 PCB congeners, plus DDE, HCB, and mirex. Results were reported as wet weight values with total serum lipids considered as a covariate, based on evidence that traditional lipid adjustment can create bias (Schisterman et al. 2005). Values below the minimal detection limit (MDL) were taken as MDL divided by the square root of 2 except as described below. Table S1 lists the congener(s) present in each peak, the congener structure, the MDL, and the percentage of subjects among the 601 whose values for that peak were below the MDL.

Diabetes was defined as having a fasting glucose > 125 mg/dL, self-report of a physician diagnosis of diabetes, or both. Thirty subjects were classified as diabetic based on self-reporting, 16 subjects had serum glucose levels > 125 mg/dL, and 65 subjects reported physician diagnosis and also had an elevated fasting glucose. A total of 111 participants were classified as diabetic, and 490 persons were classified as nondiabetic.

Initially, serum concentrations of PCB congeners were pooled into a single variable labeled “total PCBs” and the sum of concentrations of the three pesticides was considered as “total pesticides” (Model 1). For the more comprehensive evaluation, we considered each pesticide separately and grouped PCB congeners by the number of chlorines on the molecule and by the number of *ortho*-substituted chlorines (Model 2).

Because the results indicate a major role of non- and mono-*ortho* congeners (see results presented in Table 4), and the concentrations of many of these congeners were below the MDLs in a significant number of the subjects, a subset of the analyses was done after deleting all non-dioxin-like congeners for which 40% or more values were below the MDL.

To assess possible relationships between PCBs with dioxin-like structure and activity with prevalence of diabetes, we used the toxic equivalence factors (TEFs) and concentration of each dioxin-like PCB congener in our assay to determine total dioxin equivalents (TEQs), using the formula recommended by Van den Berg et al. (2006) (Equation 2):

Dioxin-like PCBs TEQ = (PCB77) × 0.0001 + (PCB105) × 0.00003 + (PCB114) × 0.00003 + (PCB118) × 0.00003 + (PCB123 + PCB149) × 0.00003 + (PCB156) × 0.00003. [2]

SAS (version 9.1; SAS Institute Inc.)was used for all statistical analysis. An alpha level of 0.05 was considered significant for all statistical tests. Logistic regression modeling was used to evaluate relationships between prevalence of diabetes and serum concentrations of POPs as well as other covariates. Odds ratio (OR) estimates were used as a measure of association. Exposure variables (i.e., serum concentrations of PCBs and pesticides) were categorized using their quartile values with sample sizes of the 1st through 4th quartile reported in each analysis. The lowest quartile was used as a referent category. In the regression analysis, the covariates of age, sex, body mass index (BMI), and total concentrations of serum lipids were treated as continuous variables. In each analysis, we also evaluated the significance of the differences of the average proportion of diabetics across the four quartiles of the model by a generalized maximum likelihood Wald χ^^2^^ test on the collection of dummy variables that define the quartile (Myers et al. 2002). Model 1 results are associations of total PCBs or total pesticides by quartiles with diabetes after adjustment for age, sex, BMI, and serum total concentrations of lipids. Results using Model 2 were obtained by adjusting all PCB congener group results for total pesticides and all individual pesticide results for total PCBs.

## Results

Table 1 shows the characteristics of the 601 study participants (224 men and 377 women, 37% and 63%, respectively) for whom all blood measurements and physical parameters were available. Mean BMI was 30.3 kg/m^2^, a value that is classified as “obese”and median BMI was 29.4, which is in the “overweight” category (NIH 1998). There were 111 participants who were diabetic based on fasting glucose levels > 125 mg/dL or who reported they were taking physician-prescribed anti-diabetic medication (41 men and 70 women). Table 2 shows wet weight serum measurements of PCBs, chlorinated pesticides, as well as their 25th, 50th, and 75th percentiles. Mean serum concentration of PCBs was 5.26 ng/g (range 1.56–49.09) and close to half of the total concentration was composed of penta- and hexachlorobiphenyls (2.56 ng/g) or di-*ortho* PCBs (2.86 ng/g).

**Table 1:** Demographic characteristics of the study population (*N* = 601).

Characteristic	Mean	STD	Min	25th percentile	Median	75th percentile	Max
Age (years)	43.9	14.3	18	33	42	54	84
BMI (kg/m^2^)	30.3	6.4	14.6	26.1	29.4	33.5	59.8
Serum glucose (mg/dL)	107.2	46.5	54.0	85.0	93.0	104.0	480.0
Total lipids (mg/dL)	594.7	151.1	270.0	487.2	578.0	675.0	1378.3
Cholesterol (mg/dL)	194.7	38.0	101.0	169.0	194.0	219.0	410.0
Triglycerides (mg/dL)	152.1	93.3	30.0	92.0	130.0	187.0	746.0

**Table 2:** Serum levels of POPs (ng/g) of study participants (*N* = 601).

Contaminant	Mean	STD	Min	25th percentile	Median	75th percentile	Max
Total PCBs	5.26	4.01	1.56	2.75	4.05	6.34	49.09
Mono/Dichlorobiphenyls^*a*^	0.27	0.07	0.24	0.24	0.25	0.26	1.36
Tri/Tetrachlorobiphenyls^*b*^	0.98	0.56	0.50	0.62	0.79	1.14	6.58
Penta/Hexachlorobiphenyls^*c*^	2.56	2.16	0.54	1.24	1.93	3.15	26.63
Hepta/Octa/Nona/Decachlorobiphenyls^*d*^	1.45	1.49	0.23	0.52	0.95	1.86	15.63
Non-/Mono-*ortho* PCBs^*e*^	1.32	1.13	0.48	0.70	0.96	1.52	14.71
Dioxin-like PCBs TEQs (×10^–5^)^*f*^	1.7	1.9	0.35	0.70	1.13	2.07	25.3
Non-dioxin-like non-/mono-*ortho* PCBs^*g*^^,^^*h*^	0.79	0.52	0.40	0.48	0.63	0.92	6.34
Di-*ortho* PCBs^*i*^	2.86	2.26	0.59	1.40	2.21	3.62	25.26
Tri-/Tetra-*ortho* PCBs^*j*^	1.04	0.79	0.40	0.56	0.80	1.25	9.07
Total pesticides	3.37	3.58	0.20	1.07	2.11	4.30	22.79
HCB	0.08	0.04	0.01	0.05	0.07	0.09	0.33
DDE	3.17	3.50	0.08	0.94	1.88	4.02	22.51
Mirex	0.12	0.15	0.01	0.03	0.07	0.16	1.67
^***a***^Mono- and di-chloro biphenyls: IUPAC# 1, 2+4, 3, 6, 7, 8, 9, 13, 15. ^***b***^Tri- and tetra-chloro biphenyls: 19, 18, 17, 24+27, 32+16; 29, 26, 25, 31, 28, 33, 53, 51, 22, 45, 46, 52, 49, 47+59, 44, 42, 71, 64, 40, 67, 63, 74, 70, 66, 56, 77. ^***c***^Penta- and hexa-chloro biphenyls: 95, 91, 92, 84, 90+101, 99, 83, 97, 87, 136, 110, 151, 144, 109+147, 123+149, 118, 134, 114, 146, 153, 132, 105, 141, 137, 130, 164+163+138, 158, 129, 128, 156. ^***d***^Hepta-, octa-, nona-, and deca-chloro biphenyls: 179, 176, 187, 183, 174, 177, 171, 201, 172, 180, 200, 170, 190, 199, 203, 196, 195, 194, 206. ^***e***^Non- and mono-*ortho* biphenyls: 1, 3, 13, 15, 7, 9, 6, 8, 29, 26, 25, 31, 28, 33, 22, 67, 63, 74, 70, 66, 56, 77, 118, 114, 105, 156. ^***f***^PCBs with our analytical methods that have dioxin-like activity: 77, 105, 114, 118, 123+149, 156. ^***g***^Non- and mono-*ortho* biphenyls that are non-dioxin-like for which 40% or more values were below the MDL: PCBs 1, 3, 6, 7, 8, 9, 13, 15, 22, 25, 26, 29, 31, 33, 56, 63, 66, and 67. ^***h***^Non- and mono-*ortho* biphenyls that are non-dioxin-like for which 40% or more values were above the MDL: PCBs 28, 70 and 74. ^***i***^Di-*ortho* biphenyls: 4+2, 18, 17, 24+27, 32+16, 52, 49, 47+59, 44, 42, 71, 64, 40, 92, 90+101, 99, 83, 97, 87, 110, 146, 153, 141, 137, 130, 164+163+138, 158, 129, 128, 172, 180, 170, 190, 194. ^***j***^Tri- and tetra-*ortho* biphenyls: 19, 53, 51, 45, 46, 95, 91, 84, 151, 144, 147+109, 123+149, 134, 132, 187, 183, 185, 174, 177, 171, 199, 203, 196, 195, 206, 136, 179, 176, 201, 200.							

Associations between serum concentrations of POPs and prevalence of diabetes were studied using logistic regression analysis and are shown in Table 3. Using Model 1 with adjustment for age, sex, BMI, and serum total concentrations of lipids, there were significant omnibus differences between the four quartiles for both total PCBs (*p* = 0.0005) and total pesticides (*p* = 0.0033). Using Model 2, only the quartiles of total PCBs, which were additionally adjusted for total pesticides, remained significant (*p* = 0.016), but none of the pairwise differences between the 1st quartile and the remaining 2nd, 3rd, and 4th quartiles reached statistical significance at the 0.05 level.

**Table 3:** Associations between diabetes and serum levels of total PCBs (101 congeners) and total pesticides (sum of HCB, DDE, and mirex) by quartiles.

Variables	Quartile 1	Quartile 2	Quartile 3	Quartile 4	Wald χ^2^ *p*-value
Total PCBs
*N* (diabetics)	155 (13)	150 (11)	153 (34)	143 (53)
Model 1
OR (95% CI)	1.0 (ref)	0.61 (0.25, 1.47)	1.84 (0.81, 4.21)	3.45 (1.41, 8.49)
*p*-Value	—	0.27	0.15	0.0069	0.0005
Model 2
OR (95% CI)	1.0 (ref)	0.49 (0.18, 1.35)	1.15 (0.41, 3.22)	2.03 (0.67, 6.16)
*p*-Value	—	0.17	0.79	0.21	0.016
Total pesticides
Total *n*, (diabetics)	149 (10)	155 (12)	145 (32)	152 (54)
Model 1
OR (95% CI)	1.0 (ref)	0.75 (0.30, 1.89)	2.61 (1.07, 6.37)	3.12 (1.12, 8.65)
*p*-Value	—	0.54	0.035	0.029	0.0033
Model 2
OR (95% CI)	1.0 (ref)	0.85 (0.31, 2.34)	2.42 (0.79, 7.39)	2.18 (0.63, 7.57)
*p*-Value	—	0.75	0.12	0.22	0.085
Note: The number (*N*) of subjects in each quartile is given and the number of subjects with diabetes is given in parentheses. Model 1–results are adjusted for age, gender, BMI, and serum concentrations of total lipids, but not for other POP groups. Model 2–results are adjusted for the other POP groups as well as age, gender, BMI, serum concentrations of total lipids. Wald χ^2^ *p*-value is for a test of differences in proportions of diabetes across the full set of quartiles.					

**Table 4:** Associations between prevalence of diabetes and quartiles of serum concentrations of PCBs grouped by number of *ortho*-substituted chlorines.

Variables	Quartile 1	Quartile 2	Quartile 3	Quartile 4	Wald χ^2^ *p*-value
Non-/mono-*ortho* PCBs
*N* (diabetics)	153 (9)	154 (13)	149 (31)	145 (58)
Model 1
OR (95% CI)	1.0 (ref)	1.03 (0.41, 2.60)	2.78 (1.17, 6.59)	5.66 (2.23, 14.34)
*p*-Value	—	0.95	0.02	0.0003	< 0.0001
Model 2
OR (95% CI)	1.0 (ref)	1.03 (0.37, 2.86)	2.37 (0.84, 6.73)	4.55 (1.48, 13.95)
*p*-Value	—	0.95	0.10	0.0081	0.0036
Di-*ortho* PCBs
Model 1
*N* (diabetics)	154 (16)	151 (9)	150 (34)	146 (52)
OR (95% CI)	1.0 (ref)	0.36 (0.14, 0.88)	1.40 (0.63, 3.11)	2.46 (1.02, 5.95)
*p*-Value	—	0.026	0.42	0.046	0.0004
Model 2
OR (95% CI)	1.0 (ref)	0.27 (0.10, 0.74)	0.79 (0.30, 2.11)	1.26 (0.43, 3.69)
*p*-Value	—	0.011	0.64	0.67	0.0059
Tri-/tetra-*ortho* PCBs
Model 1
*N* (diabetics)	152 (11)	152 (14)	150 (35)	147 (51)
OR (95% CI)	1.0 (ref)	0.97 (0.40, 2.32)	2.62 (1.11, 6.17)	4.18 (1.63, 10.70)
*p*-Value	—	0.94	0.028	0.0029	0.0015
Model 2
OR (95% CI)	1.0 (ref)	0.88 (0.34, 2/30)	1.86 (0.68, 5.06)	2.90 (0.99, 8.51)
*p*-Value	—	0.80	0.23	0.053	0.042
Note: The number (N) of subjects in each quartile is given and the number of subjects with diabetes is given in the parentheses. Model 1–results are adjusted for age, gender, BMI, and serum concentrations of total lipids, but not total pesticides. Model 2–results are adjusted for total pesticides as well as age, gender, BMI, serum concentrations of total lipids. Wald χ^2^ test evaluates the significance of the differences in proportions across all quartiles.					

Categorizing the PCB and pesticide exposure variables into quartiles in the linear logistic model analyses has the advantage of yielding results shown in easily interpretable odds ratios (Weinberg 1995). A disadvantage of categorization, however, is that the test of significance available in commercial statistical software (e.g., SAS, STATA, SPSS) of the categorized exposure effect is an omnibus test of differences between quartiles that is independent of the ordinal trend of the quartiles and could be nonlinear (Greenland 1995). To confirm the linearity of the effect in these analyses, Rothman et al. (2008, p. 410) recommend that either an ordinal sequence of the exposure quartiles (e.g., 1, 2, 3, 4) be superimposed and treated as a linear predictor, or, if available, the original continuously distributed variable be employed directly in the linear model. Thus, this is a test for linear trend. Since the original measures of PCBs and pesticides exist in a continuously distributed form, we refitted each of the 15 linear models of Tables 3–7 with the log-transformed PCBs or pesticide exposures along with the full set of covariates of Model 1. The results of these Model 1 analyses showed a positive dose–response effect for the continuous exposure variables in each of the 13 fitted models and yielded *p*-values that are commensurate (often lower) with the omnibus tests of significance of differences among odds ratios across the quartiles of exposures reported throughout this manuscript and in Tables 3–7. In the analysis of continuous exposures, the same 12 of the 13 tests were statistically significant at *p* < 0.01. The pattern of these positive dose–response relationships can also be confirmed by the number of diabetic cases relative to the total number of observations in each of the quartiles reported in Tables 3–7.

The logistic regression analysis with groups of PCBs by number of *ortho*-substituted chlorines (Table 4) showed that all groups have a significant and dose-dependent relationship (all 4th quartiles compared to 1st quartile ORs statistically significant, *p*s < 0.002) with prevalence of diabetes when adjusted only for age, sex, BMI, and total serum lipids (Model 1). When models were adjusted for these covariates, as well as total serum pesticides (Model 2), there were significant omnibus differences between quartiles for non- and mono-*ortho* (*p* = 0.0036), di-*ortho* (*p* = 0.0059) and tri- and tetra-*ortho* (*p* = 0.042) congener groups. Significant pairwise differences were observed for the 4th versus 1st quartiles of the non- and mono-*ortho* congeners {OR = 4.55 [95% confidence interval (CI): 1.48, 13.95]}. The 2nd versus 1st quartiles of the di-*ortho* congeners [OR = 0.27 (95% CI: 0.10, 0.74)] was significant but inverse while the difference between the 4th versus 1st quartiles of the tri- and tetra-*ortho* congeners [OR = 2.90 (95% CI: 0.99, 8.51)] was positive and not quite significant (*p* = 0.526).

Because the results in Table 4 implicate non- and mono-*ortho* congeners as being particularly associated with prevalence of diabetes, a separate regression analysis was done to determine if this association was due to dioxin-like or non-dioxin-like congeners. Table 5A shows results when applying the two models to dioxin TEQs, calculated based on the concentrations and TEF values for the dioxin-like PCBs present in our analysis (PCBs 77, 105, 114, 118, 123+149 and 156) as compared to the total serum concentrations of the non- and mono-*ortho* congeners (PCBs 1, 3, 6, 7, 8, 9, 13, 15, 22, 25, 26, 28, 29, 31, 33, 63, 66, 67, 70, and 74) in our analysis that do not have assigned TEFs. Serum concentrations were highly correlated between these two groups (Pearson’s correlation coefficient = 0.92, see Table S2), making it difficult to estimate independent associations. Both dioxin TEQ and non- and mono, non-dioxin-like congeners showed highly significant associations with diabetes when only adjusted for covariates. When adjusted for total pesticides, the highest to lowest quartile result for TEQs was not significant, but there was a significant *p* value (*p* = 0.010). However for non- and mono, non-dioxin-like PCBs there were significant associations for both the 3rd [OR = 2.92 (95% CI: 1.07, 7.96)] and 4th quartiles [OR = 5.01 (95% CI: 1.76, 14.24)] and a significant omnibus difference between quartiles (*p* = 0.0088).

**Table 5:** Associations between prevalence of diabetes and quartiles of serum concentrations of dioxin-like PCB TEFs and non-dioxin-like, non- and mono-*ortho* PCBs.

Variables	Quartile 1	Quartile 2	Quartile 3	Quartile 4	Wald χ^2^ *p*-value
**A. **Congeners with values below the MDL given concentrations of the MDL/square root of 2.					
Dioxin-like PCBs TEQ
*N* (diabetics)	145 (12)	163 (13)	49 (28)	144 (58)
Model 1
OR (95% CI)	1.0 (ref)	0.61 (0.25–1.46)	1.46 (0.64–3.32)	3.08 (1.27–7.49)
*p*-Value	—	0.27	0.37	0.013	0.0004
Model 2
OR (95% CI)	1.0 (ref)	0.49 (0.18–1.34)	0.93 (0.33–2.59)	1.82 (0.61–5.40)
*p*-Value	—	0.17	0.88	0.28	0.010
Non-dioxin-like PCBs
Model 1
*N* (diabetics)	155 (7)	149 (18)	150 (32)	147 (54)
OR (95% CI)	1.0 (ref)	1.87 (0.72–4.80)	3.33 (1.32–8.40)	6.01 (2.32–15.59)
*p*-Value	—	0.20	0.011	0.0002	0.0003
Model 2
OR (95% CI)	1.0 (ref)	2.00 (0.72–5.58)	2.92 (1.07–7.96)	5.01 (1.76–14.24)
*p*-Value	—	0.18	0.037	0.0025	0.0088
**B.** Congeners with more than 40% of concentrations below the MDL removed from the analysis.					
Non-dioxin-like PCBs
*N* (diabetics)	155 (10)	155 (15)	144 (27)	147 (59)
Model 1
OR (95% CI)	1.0 (ref)	1.13 (0.47–2.69)	1.86 (0.79-4.40)	4.61 (1.87–11.33)
*p*-Value	—	0.79	0.16	0.0009	0.0002
Model 2
OR (95% CI)	1.0 (ref)	1.10 (0.42–2.85)	1.49 (0.56–3.98)	3.53 (1.26–9.89)
*p*-Value	—	0.85	0.43	0.016	0.0077
Note: The number (*N*) of subjects in each quartile is given and the number of subjects with diabetes is given in parentheses. Model 1–results are adjusted for age, gender, BMI, and serum concentrations of total lipids, but not for total pesticides. Model 2–results are adjusted for total pesticides as well as age, gender, BMI, serum concentrations of total lipids. Wald χ^2^ *p*-value is the significance level of the differences in proportions of diabetes across the quartiles of each predictor variable.					

Because many of these low-chlorinated congeners frequently have concentrations below the MDLs, and because treating values below the MDL by giving them value of the MDL divided by the square root of 2 could possibly lead to error, the results for the non-dioxin-like PCBs were recalculated by removing all congeners from the analysis if 40% or more of values were below the MDL, leaving only PCBs 28, 70, and 74. These results are shown in Table 5B. There was a significant OR [3.53 (95% CI: 1.26, 9.89)] for highest to lowest quartile and a significant omnibus test across the four quartiles (*p* = 0.0077).

Table 6 shows a similar analysis now based on the numbers of total chlorines regardless of their position on the PCB molecule. Most mono and dichloro congeners had a high prevalence of values below the detection limit, and thus we did not study mono and dichloro congeners. All other PCB groups showed significant positive associations between 4th and 1st quartiles with prevalence of diabetes when models were not adjusted for other PCBs and pesticides (Model 1). After adjustment for covariates and pesticides (Model 2), there was a significant omnibus test across the four quartiles (*p* = 0.031) for tri- and tetra-chlorinated congeners, as well as a significant difference between the 4th and 1st quartiles [OR = 3.66 (95% CI: 1.37, 9.78)], and between the 3rd and 1st quartiles [OR = 3.44 (95% CI: 1.32, 8.97)]. In Model 2, neither the omnibus test nor the 4th to 1st quartiles shows significant differences for the penta- and hexachloro congeners, while for the most highly chlorinated congeners (hepta- octa-, nona- and decachlorobiphenyls) there was a significant omnibus difference among the four quartiles (*p* = 0.0071) as well as a significant negative difference between the 1st and 2nd quartiles [OR = 0.33 (95% CI: 0.11, 0.96)]. However comparisons between the 3rd and 1st and 4th and 1st quartiles did not show significant differences.

**Table 6:** Associations between prevalence of diabetes and quartiles of serum concentrations PCBs by the number of chlorines on the PCB molecule.

Variables	Quartile 1	Quartile 2	Quartile 3	Quartile 4	Wald χ^2^ *p*-value
Tri/tetrachloro-biphenyls
*N* (diabetics)	153 (7)	148 (18)	145 (39)	146 (47)
Model 1
OR (95% CI)	1.0 (ref)	1.89 (0.74, 4.84)	4.05 (1.64, 10.00)	4.58 (1.82, 11.55)
*p*-Value	—	0.18	0.0024	0.0012	0.0020
Model 2
OR (95% CI)	1.0 (ref)	1.92 (0.72, 5.09)	3.44 (1.32, 8.97)	3.66 (1.37, 9.78)
*p*-Value	—	0.19	0.012	0.0098	0.031
Penta/hexachloro-biphenyls
Model 1
*N* (diabetics)	156 (13)	147 (12)	153 (35)	145 (51)
OR (95% CI)	1.0 (ref)	0.59 (0.25, 1.42)	1.73 (0.77, 3.92)	2.74 (1.12, 6.69)
*p*-Value	—	0.24	0.19	0.027	0.0035
Model 2
OR (95% CI)	1.0 (ref)	0.47 (0.17, 1.30)	1.06 (0.38, 2.96)	1.46 (0.48, 4.46)
*p*-Value	—	0.1469	0.9088	0.5038	0.081
Hepta/octa/nona/decachlorobiphenyls	
Model 1
*N* (diabetics)	155 (15)	153 (12)	146 (32)	147 (52)
OR (95% CI)	1.0 (ref)	0.55 (0.23, 1.30)	1.47 (0.64, 3.39)	2.90 (1.15, 7.33)
*p*-Value	—	0.17	0.37	0.024	0.0013
Model 2
OR (95% CI)	1.0 (ref)	0.33 (0.11, 0.96)	0.65 (0.21, 1.97)	1.27 (0.39, 4.15)
*p*-Value	—	0.042	0.45	0.69	0.0071
Note: The number (*N*) of subjects in each quartile is given and the number of subjects with diabetes is given in parentheses. Model 1–results are adjusted for age, gender, BMI, and serum concentrations of total lipids, but not for total pesticides. Model 2–results are adjusted for total pesticides as well as age, gender, BMI, serum concentrations of total lipids. Wald χ^2^ *p*-value is the significance level of the differences in proportions of diabetes across the quartiles of each predictor variable.					

**Table 7:** Associations between prevalence of diabetes and quartiles of serum concentrations of HCB, DDE and mirex.

Variables	Quartile 1	Quartile 2	Quartile 3	Quartile 4	Wald χ^2^ *p*-value
HCB
*N* (diabetics)	214 (20)	148 (12)	119 (27)	120 (52)
Model 1
OR (95% CI)	1.0 (ref)	0.71 (0.32, 1.56)	1.78 (0.86, 3.71)	4.21 (1.89, 3.71)
*p*-Value	—	0.40	0.12	0.0004	0.0001
Model 2
OR (95% CI)	1.0 (ref)	0.66 (0.29, 1.48)	1.35 (0.59, 3.07)	2.64 (1.05, 6.61)
*p*-Value	—	0.31	0.47	0.038	0.014
DDE
Model 1
*N* (diabetics)	152 (11)	153 (11)	144 (36)	152 (53)
OR (95% CI)	1.0 (ref)	0.64 (0.26, 1.60)	2.36 (1.00, 5.57)	2.69 (1.00, 7.16)
*p*-Value	—	0.34	0.050	0.048	0.0040
Model 2
OR (95% CI)	1.0 (ref)	0.71 (0.27, 1.87)	2.12 (0.76, 5.89)	1.81 (0.57, 5.72)
*p*-Value	—	0.49	0.15	0.31	0.076
Mirex
Model 1
*N* (diabetics)	168 (14)	148 (27)	142 (32)	143 (38)
OR (95% CI)	1.0 (ref)	2.04 (0.97, 4.29)	2.20 (1.02, 4.72)	2.65 (1.20, 5.86)
*p*-Value	—	0.059	0.044	0.016	0.108
Model 2
OR (95% CI)	1.0 (ref)	2.14 (0.96, 4.77)	1.65 (0.68, 4.01)	1.36 (0.50, 3.68)
*p*-Value	—	0.062	0.27	0.54	0.26
Note: The number (*N*) of subjects in each quartile is given and the number of subjects with diabetes is given in parentheses. Model 1–results are adjusted for age, gender, BMI, and serum concentrations of total lipids, but not for total PCBs. Model 2–results are adjusted total PCBs as well as age, gender, BMI, serum concentrations of total lipids. Wald χ^2^ *p*-value is the significance level of the differences in proportions of diabetes across the full set of quartiles.					

The results of regression analysis for associations between chlorinated pesticides (HCB, DDE, and mirex) and diabetes are presented in Table 7. HCB and DDE showed significant omnibus differences between quartiles (*p*s < 0.004), as well as significant differences between the 4th and 1st quartiles in Model 1, with adjustment for only the covariates. Only HCB showed a significant omnibus difference after further adjustment for PCBs (Model 2, *p* = 0.014) with the contrast between the 4th and 1st quartiles also being significant [OR = 2.64 (95% CI: 1.05, 6.61)].

## Discussion

We have found significant associations between serum concentrations of some POPs and prevalence of diabetes in a Native American population after adjustment for age, sex, BMI, and total concentrations of serum lipids. While these results do not prove cause and effect, they are consistent with the findings and conclusions of other studies (Lee et al. 2006; Vasiliu et al. 2006; Wu et al. 2013).

When the analysis was performed on specific subgroups of POPs, we found that the significant associations were present only with certain groups of chemicals. For PCBs, the strongest significant positive associations were with the lower-chlorinated congeners (groups of non- and mono-*ortho* and tri- and tetra-chloro PCBs). This was particularly the case when comparing odds ratios between highest to lowest quartiles. We interpret these results to suggest that the strongest associations with diabetes are due to the lower-chlorinated congeners, which are less persistent in the human body.

Since previous publications have reported associations between exposure to dioxin and elevated risk of diabetes (Pesatori et al. 1998; Michalek et al. 1999; Cranmer et al. 2000; Chang et al. 2011), we determined associations with diabetes prevalence of those non- and mono-*ortho* congeners in our analysis that have assigned TEFs, as well as the non- and mono*-ortho* congeners in our analysis that do not have assigned TEF values. After adjustment for covariates and total pesticides, the OR for the dioxin-like TEQs was not significant when comparing 4th to 1st quartile [OR = 1.82 (95% CI: 0.61, 5.40)]. However even after excluding all congeners that did not have more than 40% of concentrations below the MLD, the non-dioxin-like PCBs showed a significant OR = 2.92 (95% CI: 1.07, 7.96) when comparing 3rd to 1st quartile and OR = 3.53 (95% CI: 1.26, 9.89) when comparing 4th to 1st quartile.

It was a surprise to find stronger associations with low-chlorinated non-dioxin-like PCB congeners than those with dioxin-like activity, given the results of the dioxin studies referenced (Table 5) and the demonstration in animals that coplanar PCBs impair glucose homeostasis (Baker et al. 2013). These conclusions require replication as there is low precision in some of our estimates and our methods do not monitor all dioxin-like PCBs. Positive associations with dioxin-like PCB concentrations have been reported in several publications (Everett et al. 2007; Lee et al. 2007, 2010; Persky et al. 2012). There are also numerous reports of positive associations between prevalence of diabetes and concentrations of some non-dioxin-like higher-chlorinated PCB congeners (Lee et al. 2006; Philibert et al. 2009; Tanaka et al. 2011; Gasull et al. 2012). We interpret these results to suggest that while there may be significant associations with dioxin-like PCBs, the non-dioxin-like congeners, especially those with few chlorines, contribute more to risk of diabetes.

Because all of the POPs in our analyses are lipophilic substances that migrate together to a great degree (see Table S2), a statistically significant positive association may fail to identify the specific congeners or pesticides that are responsible for the association if one does not adjust for other POPs. Earlier reports of the associations between serum POPs concentrations and diabetes have not usually adjusted for other lipophilic compounds. In our preliminary study, Codru et al. (2007) reported that after adjustment for other analytes, the association for total PCBs and PCB 153 lost significance, whereas the association for PCB 74 remained significant. PCB 74 is a tetra-chloro, mono-*ortho* congener that does not have an assigned TEF. Wu et al. (2013), using data from the Nurses’ Health Study, found a significant association between concentrations of HCB and diabetes, but not for other POPs including PCBs 118, 138, 153 and 180. Gasull et al. (2012) concluded that the association with diabetes was limited to non-dioxin-like PCBs and HCB, based on analysis of seven PCB congeners, but drew this conclusion without adjustment for other POPs.

There are similar considerations concerning the pesticides. Numerous studies have reported associations between concentrations of both DDE and HCB and diabetes (Lee et al. 2006; Everett et al. 2007; Codru et al. 2007; Rignell-Hydbom et al. 2007, 2009; Turyk et al. 2009; Ruzzin et al. 2010; Ukropec et al. 2010). However, Gasull et al. (2012) and Wu et al. (2013) reported significant associations between diabetes and PCBs and HCB, but not DDE or DDT. We found that while there was a significant odds ratio for DDE prior to adjustment for other POPs, it disappeared after adjustment for total PCBs (Model 2). Our results suggest that the association with DDE in Model 1 was due to collinearity, and that even the HCB association is weak relative to that of the low-chlorinated, non-dioxin-like PCBs.

Holford et al. (2000) reported associations between risk of breast cancer and serum PCB congeners. While they found no association with total PCB concentrations, they reported significant inverse associations of some congeners and significant positive associations with others. They discussed the issues related to collinearity, and the importance of not simply using total PCB values since there is so much information showing that different congeners and different congener groups have different health effects. We agree with these conclusions.

In a case–control study of associations between POPs and diabetes among U.S. adults 18–30 years of age, Lee et al. (2010) demonstrated non-linear dose–response relations for a number of individual PCB congeners and different pesticides. They emphasized the importance of low-dose effects, which in their study meant that they found stronger associations between the 2nd and 3rd sextiles relative to the 1st when they compared the 5th and 6th sextiles to the 1st. There is, however, a complication in interpretation of these low-dose results, in that if indeed the primary association with diabetes is stronger with lower-chlorinated non-dioxin-like congeners, these are much more rapidly metabolized than more highly chlorinated congeners (Borlakoglu and Walker 1989; Öberg et al. 2002). Thus, elevations of serum concentrations of low-chlorinated congeners are more likely to be found in the lower quartiles of exposures. These elevations in the concentration of lower-chlorinated congeners are more reflective of recent exposure, but if such exposure is continuous over time, these less persistent, lower-chlorinated congeners may still be associated with altered health status. This suggests the possibility that the apparent low-dose effect may be at least, in part, a consequence of the more rapid metabolism of lower-chlorinated congeners.

There are other implications of these findings. Lower-chlorinated PCBs are more volatile than those with high chlorination, and therefore, for low-chlorinated congeners inhalation may be an important route of exposure (Robertson and Ludewig 2011; Carpenter 2015; Lehmann et al. 2015). We have previously found a pattern of congeners in the serum of younger Mohawks that is similar to that in air, but this pattern was obscured in older individuals who had higher concentrations of more persistent PCBs (DeCaprio et al. 2005). In addition, Kouznetsova et al. (2007) reported elevations in rates of hospitalization for diabetes among individuals living near hazardous waste sites containing PCBs, and suggested that the likely route of exposure was inhalation of vapor-phase PCBs. Living near a PCB-contaminated hazardous waste site would not be expected to increase exposure via ingestion or dermal uptake, but can result in continuous inhalation of vapor-phase PCBs, although this inhalation would vary with season and temperature (Sandy et al. 2012).

This study has several limitations. Only single measurements were made of both fasting glucose level and serum PCBs and chlorinated pesticides. We relied on verbal report of medication status and disease diagnosis as well as height and weight for the study population. While participants were instructed to fast overnight before providing blood samples, this could not be objectively confirmed, and serum glucose can vary significantly in the non-fasting state. If some participants had not fasted as instructed, there could be a measurement bias impacting our findings. We do not have measurements of total serum lipids, only of total cholesterol and triglycerides. We applied the formula developed by Phillips et al. (1989) to calculate total serum lipids. Although widely used, this formula was extrapolated from a study with a relatively small number of participants who were different in terms of sex, race, and age distribution from our study population. It is also possible that there are differences between those individuals who were on physician-prescribed medication and those with previously undiagnosed high-fasting glucose (*n* = 16), but we did not examine these potential differences. The relatively small number of cases (111) resulted in imprecise estimates. There remains a possibility of residual confounding related to risk factors that were not adjusted for, such as blood pressure, serum HDL-cholesterol concentration, sedentary lifestyle, and family history.

Although we measured 101 PCB congeners, there was a significant number of values below the MDL. When we analyzed the relationship between prevalence of diabetes and PCB subgroups by number of total chlorine atoms in the molecule, only three of the group of nine mono- and di-chloro congeners showed values above the MDL. Our data on exposure to chlorinated pesticides was limited to DDE, HCB, and mirex. Previous studies have reported significant associations of other chlorinated pesticides (oxychlordane, nonachlor) with type 2 diabetes (Airaksinen et al. 2011; Lee et al. 2006).

Cross-sectional studies raise the question as to whether the results found are due to reverse causality. We consider this to be unlikely for at least two reasons. First, several previous prospective studies have reported associations between serum levels of persistent organic pollutants and subsequent development of diabetes (Turyk et al. 2009; Lee et al. 2011a, 2011b). Furthermore, if reverse causality were responsible for the results we report, one would expect this would be reflected more clearly with the more persistent congeners, not with the low-chlorinated, non-dioxin-like congeners.

There are also major strengths of our study. Our analytical method monitors more PCB congeners than most previous investigations, as well as three pesticides. The failure to measure more of the lower-chlorinated congeners in other studies may be the reason that the association of these lower-chlorinated congeners with diabetes has not been previously detected. Because higher-chlorinated PCBs are very persistent in the human body, fasting serum levels are good indicators of a lifetime of exposure. The study population had a wide range of serum PCB concentrations (mean 4.44 ng/g, range 1.54–49.09). Our analysis controlled for other major risk factors for diabetes. The direction and significance of association involving these variables is consistent with previous publications reporting associations between PCB exposure and diabetes.

The biochemical mechanisms underlying the relationship between diabetes and serum concentrations of POPs are still uncertain. Some animal studies have reported altered expression of gluconeogenic enzymes in rat liver after exposure to PCBs (Boll et al. 1998). HCB has also been reported to disrupt gluconeogenic pathways in animal models (Mazzetti et al. 2004). Various POPs activate different nuclear receptors (AhR, CAR, and PXR), which result in altered expression of genes responsible for low-grade inflammation, mitochondrial function, and fatty acid oxidation; Ruzzin et al. (2010) proposed that these actions in combination with hyperlipogenesis cause the insulin resistance syndrome. PCBs also alter the immune system (Langer et al. 2002).

## Conclusion

In this cross-sectional study, serum concentrations of lower-chlorinated PCBs and HCB showed significant association with increased prevalence of diabetes in a Native American population. These observations are consistent with the hypothesis that exposure to some but not all POPs increases the risk of developing diabetes. Because lower-chlorinated congeners are more volatile and are present in the air around PCB-contaminated sites, these results are consistent with previous studies that suggest that inhalation is an important route of exposure.

## Supplemental Material

(157 KB) PDF
