# Supplementary material for: Diabetes Prevalence in Relation to Serum Concentrations of Polychlorinated Biphenyl (PCB) Congener Groups and Three Chlorinated Pesticides in a Native American Population
Source: Environ Health Perspect. 2016 Apr 1;124(9):1376–83. doi: 10.1289/ehp.1509902 (PMC5010411; doi:10.1289/ehp.1509902)
Supplement: (157 KB) PDF [file ehp.1509902.s001.acco.pdf]

Environ Health Perspect
DOI: 10.1289/ehp.1509902

Note to readers with disabilities: *EHP* strives to ensure that all journal content is accessible to all readers. However, some figures and Supplemental Material published in *EHP* articles may not conform to 508 standards due to the complexity of the information being presented. If you need assistance accessing journal content, please contact ehp508@niehs.nih.gov. Our staff will work with you to assess and meet your accessibility needs within 3 working days.

Supplemental Material

Diabetes Prevalence in Relation to Serum Concentrations of Polychlorinated Biphenyl (PCB) Congener Groups and Three Chlorinated Pesticides in a Native American Population

Zafar Aminov, Richard Haase, Robert Rej, Maria J. Schymura, Azara Santiago-Rivera, Gayle Morse, Anthony DeCaprio, and David O. Carpenter, for the Akwesasne Task Force on the Environment

Table of Contents

Table S1. Method detection limits of PCB congeners and pesticides
Table S2. Pearson's correlation coefficients between PCBs, grouped by total number of chlorines in the molecule, and pesticides.

Table S1. Method Detection Limits of PCB Congeners and Pesticides

IUPAC#	Congenere structure	MDL(ppb)	%<MDL
1	2	0.15	100
3	4	0.04	94.5
4+2	2/2 +3	0.02	88.5
5	23	0.02	99.8
6	1	0.02	98.5
7	1	0.02	94
8	2/3.	0.02	78.9
9	25	0.02	96.8
13	4/4.	0.02	94.3
15	4/4.	0.03	90.3
17	24/2	0.03	91
18	25/2	0.02	72.2
19	26/2	0.03	89.9
22	23/4	0.01	94.3
24+27	236+26/3	0.02	88.5
25	24/3	0.01	93.7
26	25/3	0.03	89.9
28	24/4	0.02	51.7
29	2445	0.01	76.5
31	25/4	0.02	71
32+16	26/4+23/2	0.04	88.5
33	34/2	0.02	78.9
40	23/23	0.02	86.5
42	23/24	0.01	82.7
44	23/25	0.02	42.6
45	236/2	0.045	99.2
46	23/26	0.02	94
47+59	24/24+236/3	0.02	68.1
49	24/25	0.03	81.4
51	24/26	0.05	98
52	25/25	0.02	25.3
53	25/26	0.02	86.9
56	23/34	0.02	71
63	235/4	0.01	99.3
64	236/4	0.02	95.5
66	24/34	0.02	31.8

67	235/3	0.02	98.7
70	25/34	0.02	49.6
71	26/34	0.02	83.7
74	245/4	0.02	2.3
77	34/34	0.02	83.2
83	235/23	0.02	91.5
84	236/23	0.02	37.4
87	234/25	0.02	5.7
90+101	235/24+245/25	0.02	2.3
91	236/24	0.03	95.7
92	235/25	0.02	53.9
95	236/25	0.02	23.8
97	245/23	0.02	69.2
99	245/24	0.02	4.2
105	234/34	0.02	13.6
109+147	2346/3+2356/24	0.03	93.8
110	236/34	0.02	3.8
114	2345/4	0.02	40.1
118	245/34	0.02	0.2
123+149	345/24+236/245	0.02	31.3
128	234/234	0.02	69.9
129	2345/23	0.02	97.2
130	234/236	0.01	31.3
132	234/236	0.02	74.4
134	2356/23	0.01	82
136	236/236	0.03	77.7
137	2345/24	0.02	46.1
138+163+164	234/245+2356/34+236/345	0.02	0.3
141	2345/25	0.02	59.2
144	2346/25	0.02	81.2
146	235/245	0.02	8.2
151	2356/25	0.02	43.9
153	245/245	0.02	0.3
156	2345/34	0.02	16.1
158	2346/34	0.01	46.1
170	2345/234	0.02	5.7
171	2346/234	0.02	69.6
172	2345/235	0.02	42.3
174	2345/236	0.01	49.7

176	2346/236	0.01	87.4
177	2356/234	0.02	15.1
179	2356/236	0.01	80.9
180	2345/245	0.02	0.7
183	2346/245	0.01	12.3
185	23456/25	0.02	98.8
187	2356/245	0.02	2.7
190	23456/34	0.02	38.1
194	2345/2345	0.02	17.8
195	23456/234	0.02	55.1
196	2345/2346	0.01	18.3
199	2345/2356	0.01	5.3
200	23456/236	0.02	81.5
201	2346/2356	0.02	33.9
203	23456/245	0.02	21.5
206	23456/2345	0.02	37.8
HCB	HCB	0.02	1.5
DDE+85	DDE+234/34	0.02	0.2
Mirex	Mirex	0.02	16.1

Table S2. Pearson's correlation coefficients between PCBs, grouped by total number of chlorines in the molecule, and pesticides.

	Total PCBs	Total pesticides	Mono/ dichloro PCBs	Tri/tetrachloro PCBs	Penta/ hexachloro PCBs	Hepta/octa/nona/ decachloro PCBs	Non/monoortho PCBs	Dioxin-like PCBs TEQs	Non-dioxin-like mono-ortho PCBs	Di-ortho PCBs	Tri/tetra-ortho PCBs	HCB	DDE
Total pesticides	0.67<br><.0001												
Mono/dichloro PCBs	0.04<br>0.35	0.00<br>0.94											
Tri/tetrachloro PCBs	0.85<br><.0001	0.60<br><.0001	0.18<br><.0001										
Penta/hexachloro PCBs	0.98<br><.0001	0.68<br><.0001	0.00<br>0.92	0.85<br><.0001									
Hepta/octa/nona/ decachloro PCBs	0.94<br><.0001	0.58<br><.0001	-0.01<br>0.80	0.67<br><.0001	0.88<br><.0001								
Non/monoortho PCBs	0.92<br><.0001	0.67<br><.0001	0.07<br>0.07	0.93<br><.0001	0.94<br><.0001	0.76<br><.0001							
Dioxin-like PCBs TEQs	0.99<br><.0001	0.65<br><.0001	0.02<br>0.58	0.79<br><.0001	0.97<br><.0001	0.95<br><.0001	0.87<br><.0001						
Non-dioxin-like mono-ortho PCBs	0.93<br><.0001	0.57<br><.0001	0.02<br>0.64	0.70<br><.0001	0.87<br><.0001	0.98<br><.0001	0.77<br><.0001	0.92<br><.0001					
Di-ortho PCBs	0.92<br><.0001	0.67<br><.0001	-0.01<br>0.7	0.87<br><.0001	0.95<br><.0001	0.77<br><.0001	0.98<br><.0001	0.87<br><.0001	0.77<br><.0001				
Tri/tetra-ortho PCBs	0.88<br><.0001	0.64<br><.0001	0.18<br><.0001	0.97<br><.0001	0.89<br><.0001	0.71<br><.0001	0.98<br><.0001	0.83<br><.0001	0.73<br><.0001	0.92<br><.0001			
HCB	0.68<br><.0001	0.71<br><.0001	-0.04<br>0.34	0.63<br><.0001	0.68<br><.0001	0.59<br><.0001	0.68<br><.0001	0.66<br><.0001	0.57<br><.0001	0.66<br><.0001	0.67<br><.0001		
DDE	0.64<br><.0001	1.00<br><.0001	-0.01<br>0.90	0.58<br><.0001	0.66<br><.0001	0.56<br><.0001	0.65<br><.0001	0.63<br><.0001	0.54<br><.0001	0.65<br><.0001	0.62<br><.0001	0.70<br><.0001	
MIREX	0.77<br><.0001	0.37<br><.0001	0.06<br>0.16	0.60<br><.0001	0.75<br><.0001	0.75<br><.0001	0.65<br><.0001	0.78<br><.0001	0.73<br><.0001	0.66<br><.0001	0.62<br><.0001	0.40<br><.0001	0.33<br><.0001
